# Vitreous Substitutes: The Present and the Future

**DOI:** 10.1155/2014/351804

**Published:** 2014-05-04

**Authors:** Simone Donati, Simona Maria Caprani, Giulia Airaghi, Riccardo Vinciguerra, Luigi Bartalena, Francesco Testa, Cesare Mariotti, Giovanni Porta, Francesca Simonelli, Claudio Azzolini

**Affiliations:** ^1^Department of Surgical and Morphological Sciences, Section of Ophthalmology, School of Medicine, University of Insubria, Via Guicciardini 9, 21100 Varese, Italy; ^2^Endocrine Unit, Department of Clinical and Experimental Medicine, School of Medicine, University of Insubria, 21100 Varese, Italy; ^3^Eye Clinic, Multidisciplinary Department of Medical, Surgical and Dental Sciences, Second University of Naples, 80121 Naples, Italy; ^4^Department of Ophthalmology, Polytechnic University of Ancona, 60121 Ancona, Italy; ^5^Genetic Laboratory, Department of Surgical and Morphological Sciences, School of Medicine, University of Insubria, 21100 Varese, Italy

## Abstract

Vitreoretinal surgery has advanced in numerous directions during recent years. The removal of the vitreous body is one of the main characteristics of this surgical procedure. Several molecules have been tested in the past to fill the vitreous cavity and to mimic its functions. We here review the currently available vitreous substitutes, focusing on their molecular properties and functions, together with their adverse effects. Afterwards we describe the characteristics of the ideal vitreous substitute. The challenges facing every ophthalmology researcher are to reach a long-term intraocular permanence of vitreous substitute with total inertness of the molecule injected and the control of inflammatory reactions. We report new polymers with gelification characteristics and smart hydrogels representing the future of vitreoretinal surgery. Finally, we describe the current studies on vitreous regeneration and cell cultures to create new intraocular gels with optimal biocompatibility and rheological properties.

## 1. Introduction

In recent times vitreoretinal surgery has made important progress regarding instruments, drugs, and materials [1, 2]. Numerous pathologies, such as retinal detachment, diabetic retinopathy, and proliferative vitreoretinopathy, require partial or total vitreous removal [3]. Presently, temporary and permanent intraocular vitreal substitutes mainly have a structural function to ensure retinal adherence following cryo or laser retinopexy for the necessary time, to control intraocular hemorrhages, and to maintain intraocular pressure. Future polymers will interact with intraocular anatomy and physiology, as well as intraocular drug distribution [4]. One of the main challenges is the control of inflammatory and immune-system reactions that modify the stability of the vitreous substitute and the integrity and functionality of intraocular structures [5].

In this review, we examine the characteristics of the vitreous, the advantages and disadvantages of presently available tamponades, the characteristics of several vitreal substitutes studied some years ago but actually not used for several reasons, and new substances for vitreous substitution that are under research.

## 2. Characteristics of the Vitreous

The vitreous body appears as a gelatinous structure (98-99% water) filling the space between the lens and the retina, the so-called vitreous chamber. The molecular structure of the vitreous is composed mainly of hyaluronic acid and different types of collagen that create the gelatinous structure. Water is present on a bounded form to the glycosaminoglycans for about 15–20%; this ensures the stability of the vitreal structure.  Table 1 shows various molecules contained in the normal vitreous body.

Vitreous physical characteristics need to be well known in order to recognize its active role in ocular physiology, as shown in Table 2 [6]. The vitreous appears as a complex structure with its own viscoelastic properties due to a high hyaluronic acid concentration that maintains and absorbs the stress and strain of the bulb during its continuous movement during the day. The collagen-glycosaminoglycan and water frame ensure the transparency of the media, also acting as support for the vision and accommodation mechanism.

Its anatomical structure has been long studied, with recognition of its modifications due to physiological aging or pathological processes [7, 8]. The gelatinous structure is denser adjacent to the posterior hyaloid membrane (vitreous cortex) and more at the ora serrata.

The presence of active molecules allows control over inflammation, proliferation, and neovascularization, acting as a barrier to infection (bacterial not viral) [5, 9]. Finally, the vitreous body revealed its role as a repository: oxygen and nutrient as well as drugs transportation inside the eye follow definite diffusion and releasing processes [10]. These facts justify the role of the vitreous body not only as a filling substance but also as an element that has an active function on the physiology eye [11].

### 2.1. Ideal Vitreous Substitute

Since 1960, clinical and bioengineering researches have tried to find a substance that might replicate either the molecular structure of the vitreous or the physical characteristics of this gelatinous substance [12, 13]. In vitro or in vivo testing allowed evaluating not only the physical and biological parameters required to satisfy the needs of the surgeon but also the anatomy and physiology of the eye (Table 3).

We considered the fact that the vitreal substitutes could show some properties that correlated to a simple filling function (passive properties) and some properties that show interaction with intraocular structures or ocular physiology (active properties) [14, 15]. We considered as passive properties the filling action to maintain IOP, the viscoelastic characteristics to reduce shear stress on the retina, and the general inertness and biocompatibility without inflammatory ortoxic reactions [16, 17]; we considered as active properties the possibility of the new substance to interact with the biology and metabolism of the eye to permit the transportation of substances, ions, and oxygen and to maintain integrity and transparency over time [18].

An ideal testing protocol to evaluate the optimal vitreous substitute and the above properties could be summarized as follows: light transmittance, kinetics of hydration and water swelling, oscillatory and shear-stress analysis, shear-creep analysis, evaluation of solute diffusion, in vitro and in vivo biocompatibility, and degradation during injection.

These points represent the above-mentioned characteristics of the ideal long-term vitreous substitute. Numerous experimental phases must be applied to test these properties, and we are hopeful that a standardized effective model will be available in the future [15, 16].

## 3. Currently Available Vitreous Substitutes

Some of the listed substances have been known from more than 20 years, while others were developed only recently to ameliorate tolerability, tamponade effect, and stability. Here below we analyzed the advantages and disadvantages of available substances to show their current use and the short- and long-term ocular effects. Vitreal substitutes could be classified in different ways. A functional classification referred to as the surgical application is described in the literature: (i) vitreal substitutes as temporary fillers of vitreous cavity during the surgical procedure to maintain the ocular tone; (ii) vitreal substitutes used as surgical tools themselves during different phases of vitreoretinal surgery, requiring a short intraocular permanence; (iii) vitreal substitutes left inside the eye after vitreoretinal surgery with different permanence time [16, 17]. A different classification according to their molecular status, air- or gas-based and liquid, has been applied below in this paper.

### 3.1. Air

The gas used is filtered room air, composed of different gases (mainly N_2_, O_2_, CO_2_, and others at lower concentrations). Colorless and inert, it diffuses easily in the blood circulation, reducing its tamponade effects in a few days [19]. It presents a variable refractive index (approximately 1,0003). The low refractive index causes a complete reflection of the light, reducing the possibility of fundus exploration.

Air was the first gas injected into the eye. It is used in vitreoretinal surgery for retinal detachment therapy: its tamponade effect depends on the dimension and the position of the intraocular bubble, consequent to the position of the patient's head [20]. It is naturally replaced by an aqueous humor produced by the metabolism of ciliary bodies [21].

### 3.2. Gases

Sulfur hexafluoride (SF_6_), perfluoroethane (C_2_F_6_), and perfluorocarbon (C_3_F_8_) are colorless, odorless, inert, nontoxic, and expansive gas. They present a high surface tension and a specific gravity lower than water to maintain the tamponade effect [22]. When gas is injected in the vitreous cavity, it is possible to distinguish three different phases: expansion, equilibrium, and dissolution. The first phase is the result of the absorption into the bubble of nitrogen oxygen and carbon dioxide from the surrounding tissue fluid; the equilibrium phase is characterized by a balancing of the partial pressures of the media. During dissolution gases are ultimately absorbed into the bloodstream.

Sulfur hexafluoride (SF_6_) and perfluorocarbon (C_3_F_8_) are the more commonly used gases. SF_6_ expands to about the double of the volume injected within 24 to 48 hours and exerts an effect for 1 to 2 weeks; C_3_F_8_ expands to about four times its original volume within 72 to 96 hours and persists for 6 to 8 weeks. For these reasons, these gases are commercially available at a definite nonexpansive concentration (SF_6_ 20% and C_3_F_8_ 12–14%) in order to avoid errors during presurgical dilution.

Nowadays they represent the standard gases used in pneumatic retinopexy and vitreoretinal surgery, as for their longer permanence compared to the air characteristics [21, 23]. As for the air, the intraocular gas bubble has buoyancy that keeps the retina against the pigment epithelium, and this effect is greatest at the upper of the bubble. The tamponade effect is conditioned by the dimension and position of the bubble and therefore by the position of the patient's head [24, 25]. The lower refractive index, compared to corneal tissue or aqueous humor, causes almost complete reflection of light, creating fundus evaluation problematic until gas reabsorption.

Patients with intraocular gases should be advised against air travel or traveling to high altitude, since the reduction of atmospheric pressure will lead to expansion of intraocular gas bubble and cause considerable increase of intraocular pressure. At the same time they should avoid diving: the hyperbaric pressure occurring during scuba diving causes hypotony and partial globe collapse.

A great care must be applied if we expect to use these gases: if the surgery is performed in general anesthesia, dinitrogen monoxide (N_2_O) is strictly forbidden as anesthetic and analgesic due to its strong diffusion tendency. In this case the rapid vascular/eye exchange of these gases causes a rapid expansion of the intraocular bubble with severe intraocular pressure increase [26].

### 3.3. Liquids

#### 3.3.1. Saline Solution

The physical characteristics are very similar to those of the aqueous humor regarding transparency, refractive index, and density [4]. The Balanced Salt Solution (BSS, Alcon Laboratories, Randburg, USA) contains sodium chloride, potassium chloride, calcium chloride dihydrate, magnesium chloride hexahydrate, sodium acetate trihydrate, sodium citrate dihydrate, sodium hydroxide and/or hydrochloric acid (to adjust pH), and water for injection. The pH is approximately 7.5; the osmolality is approximately 300 mOsm/Kg. BSS PLUS (Alcon Laboratories, Randburg, USA) contains in addition dibasic sodium phosphate, sodium bicarbonate dextrose, and glutathione disulfide (oxidized glutathione). The reconstituted product has a pH of approximately 7.4; the osmolality is approximately 305 mOsm.

Saline solutions are used as temporary vitreous substitutes during exchange with air or liquids. They could change during intraocular permanence: proteins, cytokines, metabolites, and cells could transform this transparent fluid [27, 28] together with the aqueous fluid reaching the vitreous cavity. The solution represents a simple filling liquid, with no tamponade properties on the retina due to its low surface tension [5].

The use of different chemical compositions, like the BSS PLUS, represented a more expensive alternative. An in vivo study in rabbits has shown that BSS PLUS is more suitable than normal saline or Balanced Salt Solution for intravitreal irrigation because BSS PLUS contains the appropriate bicarbonate, pH, and ionic composition necessary for the maintenance of normal retinal electrical activity.

#### 3.3.2. Perfluorocarbon Liquids (PFCls)

They are completely fluorinated, synthetic, carbon-containing compounds that comprise exclusively fluorine-carbon bonds [29]. They are clear, colorless, and odorless; they present a density that is approximately twice that of water, low viscosity, and a refractive index similar to that of water. They are hydrophobic and lipophobic and so immiscible but they could form emulsions; they maintain the possibility of gases like CO_2_ and O_2_ to diffuse [4–30]. Three molecules are nowadays in use: perfluorodecalin (PFD), perfluoro-n-octane (PFO), and Perfluoro-tetradecahydrophenantrene that present different interface evidence when used with other fluids during surgery (PFD is at the moment the leading compound) [31].

They have been used as temporary tamponades to unfold and stabilize the retina during surgical manipulation. They have to be removed at the end of the surgical procedure [32, 33].

These substances present, if left into the eye after surgery, a retinal toxicity and intraocular inflammatory reactions, inducing the formation of epiretinal membranes and intraretinal layer disruption [34, 35].

Recently, a PFCl stained molecule has been tested to improve its surgical use with interesting results. Its usefulness will be evident during air or fluid exchange phases in which the stained tamponade will be well visible for a complete removal. Indeed, small little bubbles of PFCl adherent to the retina have been often observed a long time after the removal [36].

#### 3.3.3. Semifluorinated Alkanes

Semifluorinated alkanes (SFAs) are also known as partially fluorinated alkanes or fluorinated alkanes. These materials consist of short alkyl chains joined at one or both ends to a perfluorocarbon chain [37]. SFAs are colorless, immiscible with water, and physically and chemically inert. They present a lower viscosity and density (1.35 g/mL) than PFCls. They present solubility in PFCl, hydrocarbons, and silicone oil [38–40].

They were the first intraocular tamponades used beyond surgical time [41]. In addition, it has been demonstrated that SFAs can be successfully used also as intraoperative tools to unfold and lay down the retina. Finally they have been marketed also as biocompatible solvents for silicone oil to facilitate its removal [40].

These tamponades currently are not used owing to their tendency towards emulsification and epiretinal membrane formation and for toxic and inflammatory reactions in case of long permanence [42].

Actually they are mixed to silicone oils to form the so-called third-generation silicone oils or “heavy oils” (see the following) [43, 44].

#### 3.3.4. Silicone Oils

*Silicone Oil.* Silicone oil (SO) for ophthalmic use is a synthetic polymer belonging to the class of polydimetilsiloxanes. It presents a refractive index that is similar to the vitreous, a lower density than water, and a differing viscosity according to the type of molecule, generally 1000–5000 Centistokes (cinematic viscosity measured in Centistokes—Cs) [5].

Used in the past as an intraoperative tool to stabilize the retina and unroll the flaps of retinal tears, it is nowadays considered and recommended for long-term retinal support and tamponades, due to its chemical inertness and permanent optical transparency [45]. Its use is recommended in difficult cases as the presence of giant retinal tears, retinal detachment complicated by proliferative vitreoretinopathy [46, 47]. Due to its surface tension and hydrophobic properties it could be considered a good tamponade that depends on the position of the bubble and the patient's head (the tamponade floats over residual vitreous or water). Its intraocular presence reduces the movements and compartmentalizes cytokines and cellular factors between the anterior and posterior segment of the eye [48].

Surgical experience shows several disadvantages of long-term persistence. The complications of silicone oil use as an intraocular tamponade are mainly cataract induction, corneal toxicity, glaucoma, and so-called “silicone retinopathy” [49, 50]. A frequent modification occurring to silicone oil is emulsification. Emulsification is defined as a dispersion of fine liquid particles in another liquid medium and results from shearing forces between the two media, causing droplets to be pinched off into the other media because of surface tension. There are multiple factors affecting the emulsification of silicone oil: viscosity and physiochemical properties present an important role. Clinical research observed that the less viscous oils (1000 and 5000 Cs) tend to emulsify earlier than more viscous heavy silicone oils (see below: Oxane HD, Densiron, and HWS 46-3000) and that silicone oils containing hydroxyl and phenyl side groups emulsify earlier than purified polydimethylsiloxane. Surface-active agents (surfactants) are agents that lower the surface tension of the medium increasing its emulsification: various biological substances like blood, fibrin, and gamma globulins could act as emulsifiers and destabilize intraocular applied silicone oils [51, 52].

Macular edema represents another severe complication of silicone oil; it is present just after the exchange or increases during intraocular SO presence. This fact could take origin from different reasons: the diffusion of intraocular molecules is slowed down, reducing transport in the vitreous cavity of molecules such as oxygen and other nutrients, growth factors, and cytokines; the vitreous tamponade provides a mechanical “flotation force” at its apex against the macular region, being responsible for macula inflammation and secondary ME, especially in dynamic patients [53].

During removal procedures, problems can arise, such as hypotony and/or persistence of diffused small emulsion particles on the retina causing chronic inflammation [49, 50].

A double-fill of silicone oil and SFAs has been studied for a complete tamponade of the superior and inferior retina. The critical phase is to maintain a regular filling and to avoid the “egg effect”: in this case the separation of the two substances into two phases interrupts the correct tamponade effect [44].

*Second-Generation Silicone Oils*. Also called fluorinated silicones, they present similar characteristics to silicone oil, in particular the same viscosity and refractive index, but a higher density (greater than water) [54, 55].

They were used as vitreal substitute after surgery due to their efficacy on inferior retina tamponade. Surgical experience showed also the possibility to use them as temporary vitreal substitute to facilitate surgical procedures. Among them, the silicone fluorosilicone copolymer, a polysiloxane derivate, presents same characteristics to the fluorosilicones but due to its low viscosity facilitating injection and removal it has been used as temporary intrasurgical substitute.

All fluorosilicones present a higher emulsification rate and retinal toxicity, due probably to their high density and this fact limited their clinical use [56, 57].

*Heavy Silicone Oils (HSO)*. They have been created by the combination of silicone oil and fluorinated alkanes in a homogenous solution. Like silicone oils, they have good transparency, higher density than water, and higher viscosity. They are chemically inert, presenting an emulsification tendency less than that of silicone oils [58]. We identified four molecules: Oxane HD, Densiron 68 and 68 LV, and HWS 46-3000, as the result of the mixture of silicone oil with various SFAs. Oxane HD is a mixture of ultrapurified silicone oil (Oxane 5700) and RMN3, a partially fluorinated and hydrocarbonated olefin with a density of 1.02 g/cm^3^ and a viscosity of 3300 mPas (dynamic viscosity measured in milliPascal—mPas) [59]. Densiron 68 has been designed to take advantage of the high specific gravity of F6H8 and the high viscosity of silicone oil. The resulting solution has a density of 1.06 g/cm^3^ (higher than water) and a viscosity of 1400 mPas (substantially higher than F6H8). Densiron 68 LV is a mixture of silicone oil (siluron 1000) and F6H8 with a density of 1.05 g/cm^3^ and a viscosity of 300 mPas at 25°C [60–62]. HWS 46-3000 is a new silicone oil composed of 100,000 Cs silicone oil (45%) and F_4_H_5_ (55%) with a density of 1.118 g/cm^3^ and a viscosity of 2903 mPas [63].

They are used as long-term tamponades due to their high density and stability, in all cases where a tamponade effect on the inferior parts of the retina is necessary [64, 65].

Its removal requires strong active aspiration due to its high viscosity. The heavy SO may remain strictly adherent to the retina surface (“sticky oil phenomenon”) causing inflammation and tissue reactivity [66].

The inflammatory and toxic effects are evident on cataract induction, glaucoma, and keratopathy proving toxicity for the whole eye [67, 68].

*Magnetic Silicones*. They represent an interesting surgical experience to take advantage of the good chemical and physical properties of silicone oils. In particular, the dispersion of nanoparticles of metal (nickel, iron, cobalt, and rare metals) increases the superficial tension of the oil and therefore the tamponade effect [69].

This is carried out with the positioning of an encircling scleral magnetic band (scleral buckle). This interesting experimental project has been limited by the high toxicity of silicone oil metal dispersion on intraocular tissue [4, 69].

## 4. Experimental Substitutes

Clinical research for vitreous substitutes has essentially tried to reproduce two aspects of the original vitreous: on the one hand, a substance with the same vitreous molecular structure (simple filling function, to control elasticity and pressure of the eye), and on the other hand a structural molecule presenting its chemical and physiological properties (to assure diffusion of metabolites and gases, to allow the perfusion of drugs, and to interact actively with intraocular structures). This approach has led research toward functional biomimicry: the use of synthetic molecules that not only mimic the rheological function of the vitreous but also might interact with the intraocular structure without time-dependent degeneration or optical transparency loss [13].

### 4.1. Natural Polymers

Natural polymers, such as hyaluronic acid (HA) and collagen, have been evaluated as the basis for vitreous substitutes. As the main components of the vitreous, they present great biocompatibility. Hyaluronic acid and its derivatives are present in various formulations for ocular use, but due to the short degradation time they cannot be used as intraocular tamponades. Collagen derivatives, such as gelatine, polygeline, and methylated collagen types I-II, as well as chitosan (a natural crustacean product), have been studied as structural polymer proteins for experimental vitreous substitutes with poor results [4, 5, 70, 71].

The intraocular gel hylan, created using cross-linked molecules of sodium hyaluronate formaldehyde, divinyl sulfone, and gellan molecule, could represent an interesting short-term vitreal substitute for its stability and composition. Its excessive water solubility made it at the moment not available for clinical experiments [66].

The above-described vitreous substitutes are not effective due to the tendency of the molecules towards degradation, their low viscosity, and poor tamponade effect [72, 73].

A promising approach, compromising the biocompatibility of HA and the duration of a complex polymer, is the application of dihydrazide photo-cross-linking reaction. This type of cross-linked HA presents good transparency, viscosity, and tamponade effect due to its hydrophilic properties. Degradation time is quite long (more than 4 weeks) [74]. The advantages of this substitute are the limited tissue inflammatory and toxic reaction [75]; the disadvantage is already the short time of degradation (from 60 to 150 days) due in part to the injection procedure that alters the gel molecular structure reducing the integrity and stability. The cross-linking processes by in situ gelification [76] and the intraocular injection of cellular components to actively produce polymer matrix represent a possible solution of this problem.

### 4.2. Hydrogels

Polymeric and Smart Hydrogels represent the new class of experimental vitreal substitute [77].

These substances are hydrophilic polymers that form a gel network when cross-linked and are capable of swelling by absorbing several times their own weight in water [78]. They present good and stable transparency, good biocompatibility, and viscoelastic properties like the vitreous body, mimicking its biofunctionality, yet they have different chemical and physical properties [4, 79]. Both types of molecule are synthetic polymers with different characteristics. In particular, Smart Hydrogels are able to respond to the environment and to external physical stimuli. Their characteristics determine long-term vitreous stability without toxic effects. The passive action of these molecules as tamponades is coupled with the active action as drug releasers or exchangers to ensure therapeutic and clinical effects [80].

Hydrogel molecules have been developed and carefully selected not only owing to their chemical-physical properties, but also due to their possible toxicity [77]. They represent the first biomaterials ever synthesized for human use and have various clinical applications.

Here we list the principal molecules, showing their advantages and disadvantages; several of these ones have been discarded due to toxicity or unable characteristics. We underline that in vivo research is as yet applied only to animal models [81].

- Poly(vinyl alcohol) (PVA): it is selected for its good optical properties making it a valid vitreous substitute; it is indistinguishable from the vitreous during the initial months following injection. PVA presents good biocompatibility and rheological properties. Adding different chemical reactants, in particular trisodium-triphosphate cross-linking agent, the molecule changes and improves its properties, particularly its rheological characteristics and diffusion behavior [82]. Further studies must be carried out on its ability to act as a retinal tamponade [83].
- Poly(1-vinyl-2-pyrrolidone) (PVP): it is the first studied element for vitreous substitution. This molecule is the result of the polymerization of 1-vinyl-2-pyrrolidone with different cross-linking agents [84]. Experimental research has created several molecules of PVP, presenting a density and viscosity similar to the human vitreous, but with intraocular reactivity [85]. Transient or permanent vitreous opacification is the most frequent adverse event, as well as inflammation with vacuoles and granules, indicating early PVP degradation due to phagocytosis [86]. Further studies are underway to evaluate more tolerable and more stable PVP polymers [87, 88].
- Polyacrylamide (PAA): it is created by the polymerization of toxic acrylamide by cross-linking agents (once injected into the vitreous cavity after the monomer) [89]. Experimental PAA polymers have been created with a disulfide cross-linking agent to produce highly purified molecules [90]. PAA presents similar density and viscosity to the vitreous, as well as good biocompatibility and long-term stability. Better results are expected in the future. Severe complications such as ocular inflammation and vitreous opacification were reported on the first experimental phases of these materials.
- Copoly(acrylamide) (CPA): it is a variant of PAA presenting better gelification properties, acquiring polymerization after reduction of disulfide cross-linking bridges [90]. With the same refractive index and viscoelastic parameters of the vitreous, as well as good biocompatibility, it seemed to be a valid long-term substitution. The tested molecule showed clinical suitability and lack of significant ocular toxicity.
- Poly(glyceryl methacrylate) (PGMA): this polymer is nowadays excluded from research owing to its fragmentation upon injection [4]. The dehydrated molecule has been tested by direct intraocular positioning: in contact with intraocular fluids the molecule swells and became the vitreal substitute. The experimental evaluation found this process too slow and not effective for clinical use [91]. Although it has good biocompatibility and excellent physical properties, the molecule did not become clinically available [92].
- Poly(2-hydroxyethyl methacrylate) (PHEMA): this polymer presents solid features. Experimental research has shown good inertness to degradation and inflammatory reactivity [89]. Because of its solid feature, it caused important surgical difficulties for its implantation, so it was considered unsuitable for clinical use [93, 94].
- Poly(2-hydroxyethylacrylate) (PHEA): this hydrogel presents excellent physical properties similar to those of the human vitreous. Due to reported inflammatory reactions following injection, cataract, glaucoma, and the formation of fibrous membranes it was abandoned for human clinical research [4, 5].
- Hydroxypropyl methylcellulose (HPMC): it presents good physical-chemical properties as well as good biocompatibility [95]. Different experimental polymers have been studied, varying the molecular weight [96]. Researchers have tried to reduce intraocular degradation time, but as of today it is not yet available as a long-term vitreal substitute [97].
- Pluronic F127 (p-F127): it is a thermoreversible gelatin. It could form a gel at 21°C but it shows severe retinal toxicity making it unsuitable for clinical use [98, 99].
- Silicone gel: it is a hydrophobic polymer that maintains good intraocular transparency and cohesiveness. Its poor tamponade effect has deemed it unsuitable as an intraocular retinal surgical tool [100].
- ADCON hydrogel: it is a polymer of proteoglycan esters in porcine gelatine and it is already used in neurosurgery. This hydrogel is highly biocompatible but presents potential retinal toxicity and postoperative inflammation. It is unsuitable for ocular use [101].
- Poly(vinyl alcohol methacrylate) (PVA-MA): this polymer contains a photoinitiator that forms a gel network after irradiation at 365 nm. The degree of gelification can be regulated by polymer concentration and light intensity. PVA-MA properties must be tested in vitro and in vivo to evaluate vitreous biomimicry and biocompatibility [102].

Beyond all different experimental problems described above, a critical phase during physical tests for all these polymers was the injection through small caliper needles, a critical phase for clinical use. The shear stress of the needle during intraocular injection causes a loss on elasticity and a fluidification of the preformed molecules of hydrogel, due to the rupture of polymeric chains [77].

To resolve this criticality, hydrogel could be injected in an aqueous state and transformed into a gel in situ by light exposure or air oxidation, thanks to cross-linking processes. According to the different polymers, the liquid hydrogel could reach final gelification with defined elasticity and swelling in the presence of a photoinitiator or a disulfide cross-linker. In particular, PVA-MA is sensitive to a defined UVA wavelength, not yet applicable in eye surgery; differently, CPA is injected on a reduced form, sensitive to air oxidation for the gelification process [102].

Smart Hydrogels present similar characteristics compared to the polymeric hydrogels, but they have more interactive properties with the environment, such as glucose-, glutathione-, and pH-dependent activity and reactivity to light, pressure, and electric fields. These properties mean that these molecules could interact with retinal tissue, injected drugs, lasers light, or other chemicals and physical stimuli. These interactions induce an increased gelification, better drug diffusion, and increased gel expansion [103–106]. Little information regarding their toxicity or inflammatory action is available at present [107, 108]. Thermosetting gels are Smart Hydrogels that modify their status according to temperature (e.g., WTG-127 gel) [109]. This is important for the gelification status and viscosity, for their injection and handling. The disadvantage of this molecule is its reduced degradation time and its tendency to drift under the retina in the presence of tears before complete gelification [105].

All these molecules, as we described above, could actually cause adverse reactions of the ocular tissues at different stage, such as inflammation, phagocytosis, and vacuolization, due to molecular degradation and immune reaction. One of the major challenges is to make these molecules more and more compatible with the immune and biological systems [103].

Despite the above reasons, hydrogels seem to be the best candidates for vitreous substitution. They present all the characteristics needed to mimic the physical-chemical behavior of the vitreous, plus its biological function. We need to perform more experimental evaluations to tailor density and rigidity, as well as degradation times to match those of the natural vitreous [74, 75, 78].

### 4.3. Transplant and Implants

Many years ago, some authors described the first attempt to transplant vitreal tissue [110–112]. They observed that, if correctly stored, the vitreous body could maintain its structure and also its enzymatic properties, as described in the literature [8, 9, 11]. The implanted tissue showed a degradation time on the host, with a low inflammatory reaction and interesting surgical results on 40% of patients. Cataract, glaucoma, and more severe adverse events until ocular atrophy were described [110–112].

Regarding implants, bioengineering studies have shown interesting results in the use of artificial capsular bodies, made of silicone rubber elastomer and filled with a saline solution, silicone oil, controlled using a valve system. The system was described as being well tolerated on an experimental model. This foldable capsular vitreous body (FCVB) presented good mechanical, optical, and biocompatible properties in vitro and in vivo and has been seen to be effective as a vitreous substitute in the treatment of severe retinal detachment. The presence of a filled capsule reduces the toxic effect, such as intraocular toxicity, emulsification, high IOP, and keratopathy [113]. A new polyvinyl alcohol (PVA) filling molecule has been evaluated in recent studies on a rabbit model. The 3% concentration showed the best results in rheological, physical, and cytotoxicity tests. This type of approach combines the efficacy of hydrogel as a vitreous substitute to the presence of an implant as an isolator that could reduce degradation time. In the PVA-FCVB rabbit implanted eyes, the structure of the retina was intact at 90 days postoperatively (a lensectomy was performed in all eyes due to frequent cataract induction of the implant); at 180 days retinal disorders were reported due to long-term capsule-induced mechanical pressure to the retina [114]. The advantages of this type of approach have also been reported on a therapeutic target: several nanometer wide apertures are available on the implanted capsule, so drugs could be added to the hydrogel and long-term release could be performed [115, 116].

### 4.4. Vitreous Regeneration

The challenge to create a new vitreous with the critical 3D structure might be very interesting and for this purpose different studies were performed. Controlled hyalocytes proliferation with specific growth factors (bFGF stimulates and TGF-B1 inhibits) and the production of HA with related components were evaluated [117, 118]. Reverse transcriptase polymerase chain reaction (RT-PCR) analyzed and compared the expression profiles for several genes in the human vitreous tissue-derived cells. The regulation of hyaluronan production in response to cytokine stimulation, the expression of hyaluronan synthase isoforms using RT-PCR, and hyaluronan production using enzyme-linked immunosorbent assay (ELISA) were also investigated[119].

## 5. Conclusions

The vitreous is a fundamental component of the eye. It has filling functions and extremely active properties on the stability and metabolism of the retina complex. Current long-term vitreous substitutes are clinically largely used but present some disadvantages. Many studies evaluated the possibility to realize the ideal vitreal substitute: long-term persistence and good biocompatibility to maintain transparency and integrity. Polymeric hydrogels have shown suitable characteristics with great variability of chemical composition: ideal substitution must be performed correctly, and experimental research is advancing.

## Figures and Tables

**Table 1:** Biochemical composition of the vitreous.

Subgroups	Molecule	Action
Protein	Albumin (40%)	
	Iron binding protein (30%) like transferrin	Protective effect to reduce iron toxicity
	Collagens	Structure of the vitreous
	Type II (60–70%)	
	Type IX (25%)	
	Type V/IX (10–25%)	
	Type IV (<10%)	

Glycosaminoglycan	Hyaluronic acid (66–115 microgram/mL concentration)	Determine the vitreous body viscosity
	Chondroitin sulfate	Major component of extracellular matrix
	Versican	
	Type IX collagen	
	Heparan sulfate	It maintains adequate spacing between the collagen fibrils

Metabolites	Glucose	To support the enzymatic activity
	Lactic acid	
	Ascorbic acid	Neovascularization inhibitor Increase proliferation of hyalocytes Potent antioxidant
	Amino acids	Metabolic cells maintenance
	Fatty acids unsaturated (50–55%)	Metabolic cells maintenance
	Prostaglandins (100 picogram/mL)	Cells regulation
	PGE2	Cells regulation
	PGF2alpha	Cells regulation
	Prostacyclin	Cells regulation
	Thromboxane	Cells regulation

Cells	Hyalocytes	Vitreous matrix creation and maintenance
	Fibrocytes/fibroblasts	Vitreous matrix creation and maintenance
	Macrophages	Cells and matrix regulation and degradation
	Enzymes and metabolic activity: ACE	Cells regulation

**Table 2:** Physical characteristics of the vitreous.

Physical characteristics of the vitreous	
Weight	4 g
Density	1.0053–1.008 g/cm^3^
Refractive index	1.3345–1.3348
Viscosity	300–2000 cP
pH	7.0–7.4

**Table 3:** Characteristics of the ideal vitreous substitute.

The ideal vitreous substitute	
Mimic the native vitreous	
Be easily manipulable during surgery	
Have similar viscoelastic proprieties	
Be clear and transparent	
Have refractive index and density similar to native vitreous	
Be biologically and chemically inert	
Be hydrophilic and insoluble in water	
Be able to maintain the IOP within a physiologic range and support the intraocular tissues in proper position	
Allow movement of ions and electrolytes and maintain the concentration of certain substances (oxygen, lactic acid, and ascorbic acid)	
Be clear	
Not induce toxic reactions	
Be biocompatible	
Be easily available, stable, and injectable through a small syringe	
Be able to maintain its light transparency post-op without undergoing opacification	
